# Genetically engineered crops for sustainably enhanced food production systems

**DOI:** 10.3389/fpls.2022.1027828

**Published:** 2022-11-08

**Authors:** Mughair Abdul Aziz, Faical Brini, Hatem Rouached, Khaled Masmoudi

**Affiliations:** ^1^ Department of Integrative Agriculture, College of Agriculture and Veterinary Medicine, United Arab Emirates University, Al−Ain, Abu−Dhabi, United Arab Emirates; ^2^ Biotechnology and Plant Improvement Laboratory, Centre of Biotechnology of Sfax, University of Sfax, Sfax, Tunisia; ^3^ Michigan State University, Plant and Soil Science Building, East Lansing, MI, United States

**Keywords:** GM crops, genome editing, sustainable agriculture, food production, environmental constraints

## Abstract

Genetic modification of crops has substantially focused on improving traits for desirable outcomes. It has resulted in the development of crops with enhanced yields, quality, and tolerance to biotic and abiotic stresses. With the advent of introducing favorable traits into crops, biotechnology has created a path for the involvement of genetically modified (GM) crops into sustainable food production systems. Although these plants heralded a new era of crop production, their widespread adoption faces diverse challenges due to concerns about the environment, human health, and moral issues. Mitigating these concerns with scientific investigations is vital. Hence, the purpose of the present review is to discuss the deployment of GM crops and their effects on sustainable food production systems. It provides a comprehensive overview of the cultivation of GM crops and the issues preventing their widespread adoption, with appropriate strategies to overcome them. This review also presents recent tools for genome editing, with a special focus on the CRISPR/Cas9 platform. An outline of the role of crops developed through CRSIPR/Cas9 in achieving sustainable development goals (SDGs) by 2030 is discussed in detail. Some perspectives on the approval of GM crops are also laid out for the new age of sustainability. The advancement in molecular tools through plant genome editing addresses many of the GM crop issues and facilitates their development without incorporating transgenic modifications. It will allow for a higher acceptance rate of GM crops in sustainable agriculture with rapid approval for commercialization. The current genetic modification of crops forecasts to increase productivity and prosperity in sustainable agricultural practices. The right use of GM crops has the potential to offer more benefit than harm, with its ability to alleviate food crises around the world.

## 1 Introduction

Agriculture faces severe challenges for delivering food and maintaining nutritional security through sustainable practices. relation to the concept of sustainability, sustainable agriculture is defined as a system of growing crops for the short and long-term period without damaging the environment, society, and the economy for the present and future generations (Tripathi et al., 2022). The main goals of sustainable agriculture are to produce high yield of healthy crop products, efficiently use the environmental resources with minimal damages, enhance the quality of life within the society through the just distribution of food, and provide economic benefits for the farmers (Tseng et al., 2020). These goals have become a prominent issue of discussion in agriculture in the past few years and have been recognized widely in scientific communications, since it is difficult to produce large amounts of food with minimal environmental degradation.

However, there has been a remarkable breakthrough in the field of agriculture through plant genetic modification. Plant biotechnology has generated products that helped agriculture sector to achieve enhanced yields in a more sustainable manner. It has witnessed an increase in the production capacity that is as huge as it was during the period of the green revolution in the early 70’s (Raman, 2017). A genetically modified (GM) crop is defined as any plant whose genetic material has been manipulated in a particular way that does not occur under natural conditions, but with the aid of genetic techniques (Sendhil et al., 2022). Agriculture is the first sector that invested heavily in the use of genetic modifications (Raman, 2017). The massive experiments in agricultural biotechnology have enabled the development of suitable traits in plants for food production. The employment of genetic tools for the introduction of a foreign gene, as well as the silencing and expressing of a specific gene in plants, have brought a dramatic expansion of GM crops (Kumar et al., 2020). It has led to the propagation of crops that are disease resistant, environmental stress tolerant, and have an improved nutrient composition for consumers (Batista et al., 2017).

The techniques for the improvement of plants for food production have been undertaken since the humankind stopped migration and relied on agriculture for their survival. At present, more advanced molecular tools are developed for specific genetic manipulation of crops than the conventional methods. Genome editing is the process of making targeted improvements to a plant’s genome, specifically within plant’s own family (Kaur et al., 2022). Its precision in changing almost any desired location in the genome makes it discrete from other breeding methods. Most of the changes that are made through genome editing occurs naturally within the plants, through traditional breeding or evolution (Graham et al., 2020). However, through genome editing such results are obtained within years rather than decades. With this method, there is no addition of foreign genes, and it is more accurate and predictable than earlier techniques of plant genetic modification (Kaur et al., 2022).

In the twenty-first century, the genetic modification of crops is considered a potential solution for achieving the goals of sustainable agriculture (Oliver, 2014). However, the use of GM crops has raised complex issues and dilemmas related to their safety and sustainability. There have been several debates which have led some countries to contest the use, cultivation, and commercialization of GM crops (Kikulwe et al., 2011). Specifically, the majority of European and Middle Eastern countries have imposed full or partial limitations on the commercialization of GM crops. Regulatory approval for the commercialization of GM crops is hampered by poor communication and awareness brought about by consumer mistrust (Mustapa et al., 2021). Moreover, the difficult process of completing risk assessments and meeting biosafety regulations, has only compounded the existing mistrust of GM crops, based on ethics, history and customs.

Nevertheless, because the GM crops are considered as good candidates for sustainable food production, it is imperative to perform the risk assessment of any developed GM crop, exploring their negative and positive consequences for the current agricultural developments. In this regard, the goal of the present study is to evaluate the use of genetic manipulation and genome editing of crops for overcoming the global food challenges in a sustainable manner. It aims to review current knowledge of GM crops, the concerns and dilemmas associated with them and provides appropriate solutions to overcome them. The study further delivers several perspectives on their incorporation into sustainable food production systems and eliminate the mistrust placed on GM crops for the achievement of Sustainable Development Goals (SDGs).

## 2 Developmental pathway of GM crops over the years

**Figure 1:**
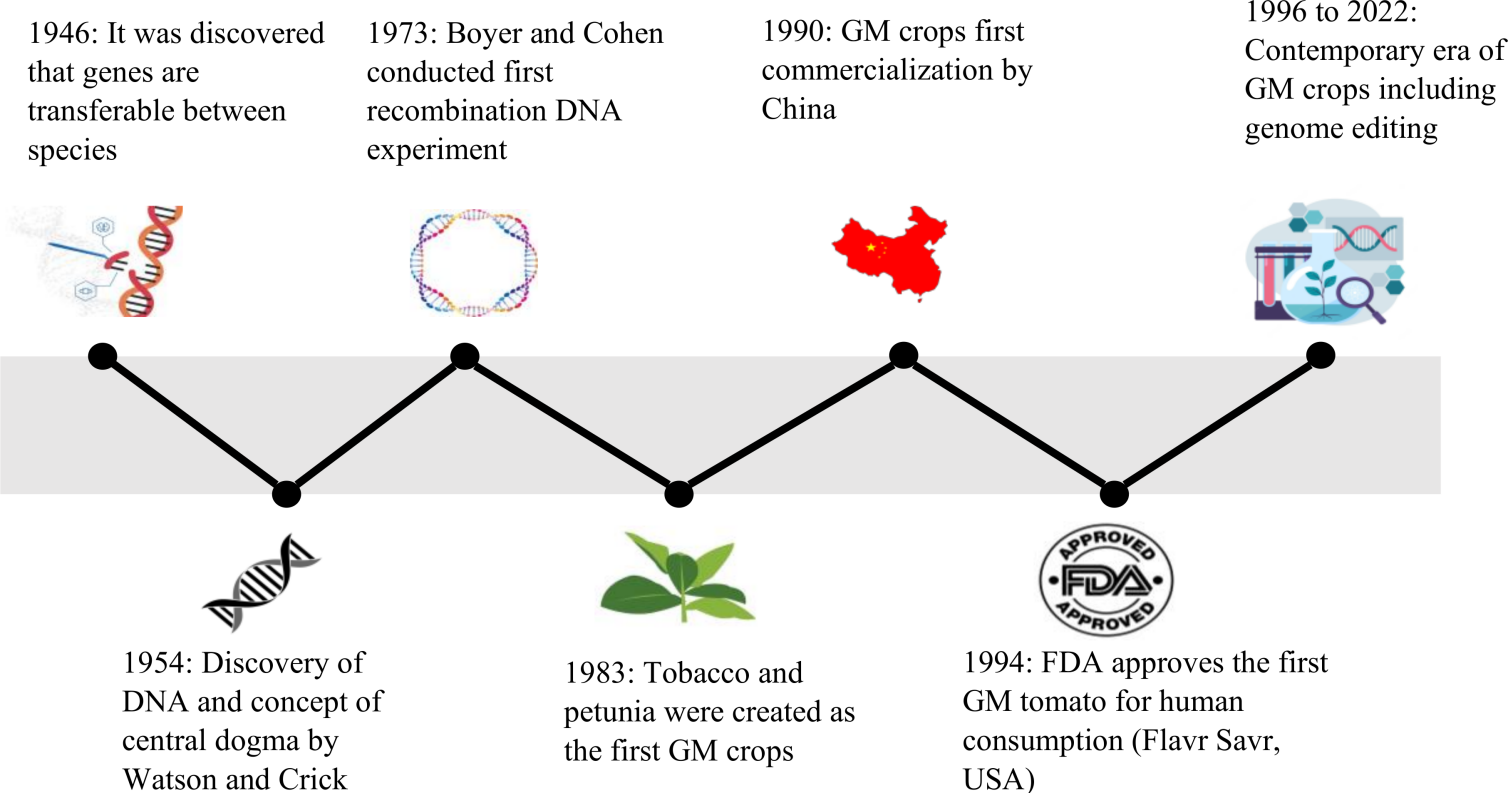
Timeline of various events from the discovery of genes being transferable during 1946 leading to the contemporary era of advanced tools for developing GM crops.

The genetic modification of plants dates back approximately 10,000 years with the practice of artificial selection and selective breeding. The selection of parents with favorable traits and their utilization in breeding programs has facilitated the introgression of these traits into their offspring’s (Raman, 2017). For instance, artificial selection of maize out of weedy grasses having smaller ears and less kernels, has resulted in the generation of edible maize cultivars (Doebley, 2006). In 1946, the advancement leading to contemporary genetic modification took place, with the scientist’s discovery of genetic material being moveable between various species (**Figure 1**) (James, 2011). This was accompanied with the identification of the double helical DNA structure and concept of the central dogma in 1954 by Watson and Crick (Cobb, 2017). Successive advances in the experiments by Boyer and Cohen in 1973 that included the extraction and introduction of DNA between various species resulted in the engineering of the World’s first GM organism (Cohen et al., 1973). In 1983, antibiotic resistant tobacco and petunia, first GM crops, were auspiciously developed by three independent scientists (Fraley, 1983).

In 1990, GM tobacco plants that were resistance to tobacco mosaic virus (TMV) were first commercialized by China (Wu and Butz, 2004). In 1994, Food and Drug Administration (FDA) approved the Flavr Savr tomato (Calgene, USA) as the first GM crop for human consumption (Vega Rodríguez et al., 2022). The antisense technology was used to genetically modify this tomato plant by interfering the production of the enzyme polygalacturonase, major enzyme responsible for pectin disassembly in ripening fruit, that retarded its ripening and protected it from rot (Bawa and Anilakumar, 2013). Several transgenic plants were approved since then for expansive production in 1995 and 1996. For instance, transgenic cantaloupe Charentais melons expressing an antisense ACC oxidase gene were developed to block their ripening process (Ayub et al., 1996). Some of the GM crops that received initial FDA-approval included cotton, corn and potatoes (modification of *Bacillus thuringiensis* (Bt) gene, Monsanto), Roundup Ready soybeans (resistance to glyphosate, Monsanto), and canola (increased oil production, Calgene) (Bawa and Anilakumar, 2013). At present, the genetic modifications are performed on various cereals, fruits, and vegetables that includes rice, wheat, strawberry, lettuce, and sugarcane. The genetic modifications are also carried out to increase vaccine bioproduction in plants, improved nutrients in animal feed, and for conferring environmental stresses such as salinity and drought (Kurup and Thomas, 2020).

### 2.1 Method of genetic modification of crops

**Figure 2:**
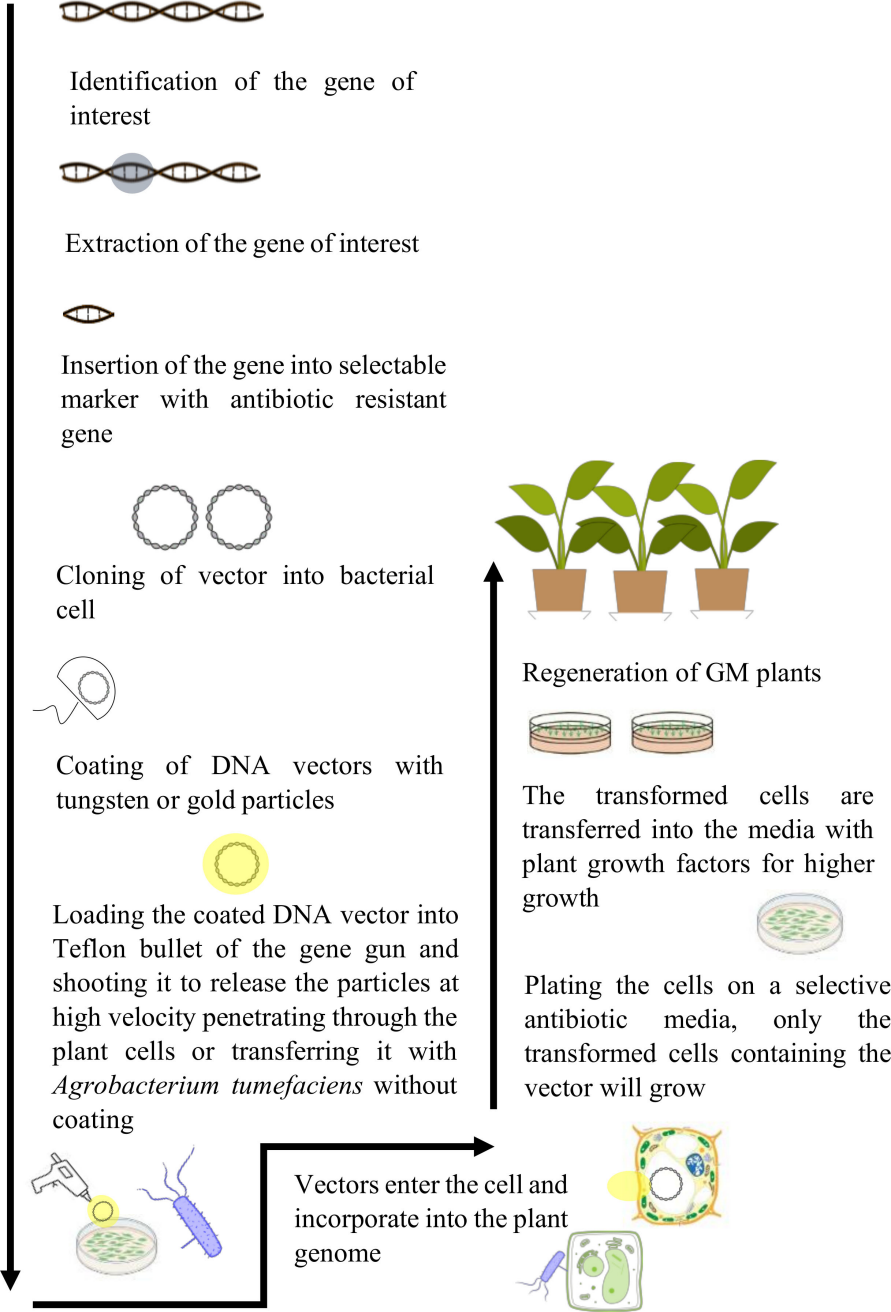
Illustration of the process of genetic modification of crops. It involves the identification of gene of interest, its isolation, and insertion into the genome of a desired plant species. The modified plants are regenerated and used for commercialization.

The creation of a GM crop is a complex phenomenon that involves several steps, from the identification of the target gene to the regeneration of transformed plants (**Figure 2**).

#### 2.1.1 Target gene identification

Developing a GM plant requires the determination of the gene of interest for a particular trait such as drought tolerance gene that is already present in a specific plant species (Snow and Palma, 1997). The genes are identified using the available data and knowledge about their sequences, structures, and functionalities. In case of an unknown gene, a much laborious method will be used, such as map-based cloning. The gene of interest is isolated and amplified using the Polymerase Chain Reaction (PCR). It allows the desired gene to be enlarged into several million copies for the gene assembly (Schouten et al., 2006).

#### 2.1.2 Cloning of the gene of interest and its insertion into a transfer vector

After several copies of genes are attained, it is inserted into a construct downstream a strong promoter and upstream a terminator. This complex is then transferred into bacterial plasmid (manufacturing vectors), allowing for the duplication of gene of interest within the bacterial cell (Zupan and Zambryski, 1995). The DNA construct with the gene of interest is introduced into the plants *via Agrobacterium tumefaciens* or gene gun (particle bombardment) (Lacroix and Citovsky, 2020).

#### 2.1.3 Modified plant cells selection and plant regeneration

When using antibiotic resistance as a selectable marker gene, only transformed plant cells survive and will be regenerated to entire plant using different regeneration techniques (Ibáñez et al., 2020). Several genetic analyses are performed for the determination of insertion and activation of the gene of interest and its interaction with different plant pathways that may cause unintended changes in the final traits within the plants (Shrawat and Armstrong, 2018).

The transformed plants are introduced into the field conditions and risk assessments are performed for their environmental and health impacts (Giraldo et al., 2019). Nonetheless, plants with foreign genes have remained in the scrutiny of society for crop production. To overcome these concerns related to transgenic crops, newer biotechnological techniques, such as cisgenesis and intragenesis, are developed as alternatives to transgenesis (Holme et al., 2013; Kumar et al., 2020). In these methods, genetic material used for trait enhancement are from identical or related plant species with sexually compatible genes.

Besides these techniques, genome editing tools has enabled the plant transformation with ease, accuracy, and specificity. Some of these methods including Zinc Finger Nucleases (ZFNs), Transcription Activator-Like Effector Nucleases (TALENs), and Clustered Regularly Interspaced Short Palindromic Repeats (CRISPR)/Cas system, were directed towards the concerns related to the unpredictability and inefficiency of traditional transgenesis (Bhardwaj and Nain, 2021). These tools are set for developing enhanced plant varieties through accurate modification of endogenous genes and site-specific introduction of target genes.

### 2.2 Status of GM crops

**Figure 3:**
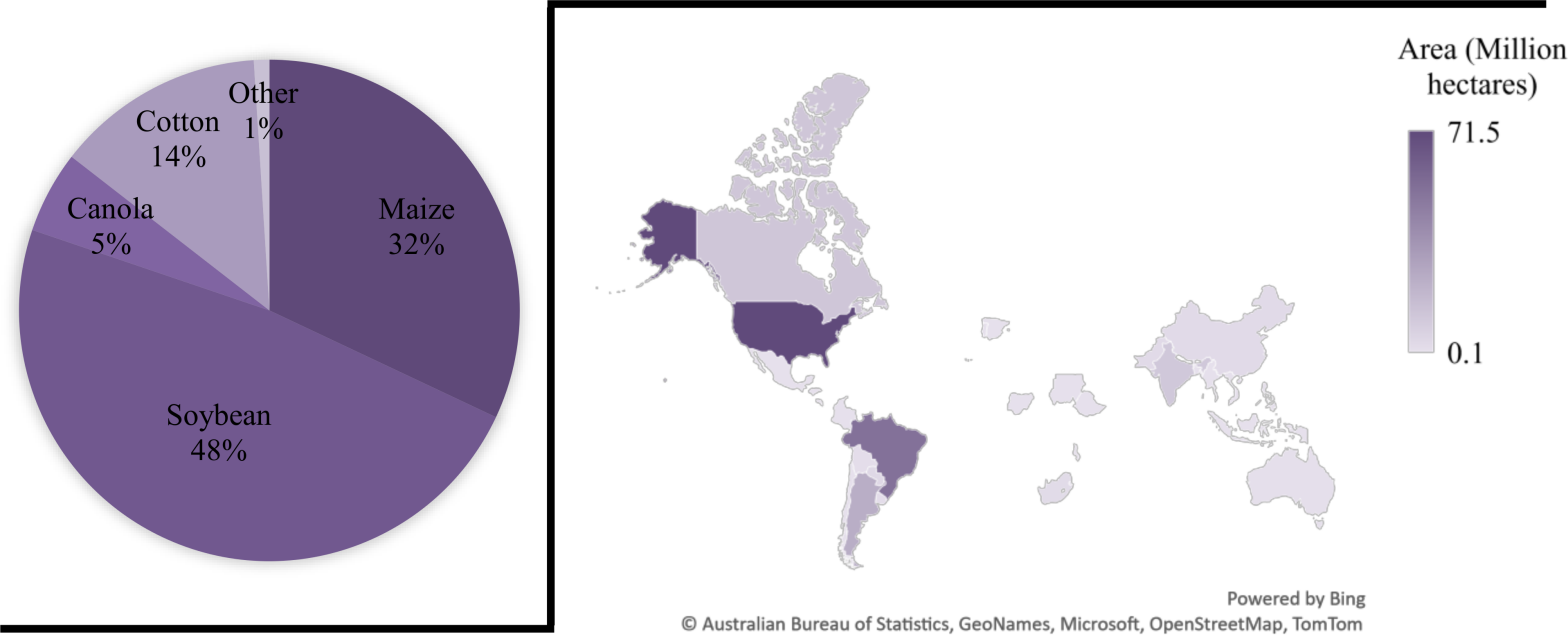
Percentage of Globally adopted GM crops and their production area (hectares) in various countries. The largest proportion of GM crops grown are soybeans (48%) and the USA covers a substantial area of 71.5 Mha with different GM crops.

The global production status of GM crops has increased between the year 1996 to 2019, from 1.7 to 190.4 million ha with approximately 112-fold increase (**Table 1**; **Figure 3**) (ISAAA, 2019). Subsequently, a large increase occurred in the commercialization of GM crops at an elevated rate in the history of present-day agriculture. Currently, the world’s largest GM crops producer is USA with 71.5 Mha (37.5%), with GM cotton, maize, and soybean accounting for 90% of its production (ISAAA, 2019). Brazil was the second largest GM crops producer with 52.8 Mha (27.7%) and Argentina was the third largest producer with 24 Mha. Canada and India were fourth and fifth largest producers with 12.5 and 11.9 Mha, respectively (ISAAA, 2019).

**Table 1:** The proportion of area covered and common GM crops in various parts of the world.

No.	Continent	Country	Area (Mha)	Common GM crops
1	North America	United States	71.5	Cotton, papaya, alfalfa, sugar beet, rapeseed, soybean, maize, and squash
2	South America	Brazil	52.8	Soybean, cotton, and maize
3	South America	Argentina	24	Cotton, soybean, and maize
4	North America	Canada	12.5	Soybean, sugar beet, rapeseed, and maize
5	Asia	India	11.6	Cotton
6	South America	Paraguay	3.8	Maize, soybean, and cotton
7	Asia	China	2.9	Tomato, sweet pepper cotton, papaya, and poplar
8	Asia	Pakistan	2.8	Cotton
9	Africa	South Africa	2.7	Cotton, soybean, and maize
10	South America	Bolivia	1.3	Soybean
11	South America	Uruguay	1.3	Maize and soybean
12	Asia	Philippines	0.6	Maize
13	Australia	Australia	0.8	Rapeseed and cotton
14	Asia	Myanmar	0.3	Cotton
15	Africa	Sudan	0.2	Cotton
16	North America	Mexico	0.2	Soybean and cotton
17	Europe	Spain	0.1	Maize
18	South America	Colombia	0.1	Cotton and maize
19	Asia	Vietnam	0.1	Maize
20	North America	Honduras	< 0.1	Maize
21	South America	Chile	< 0.1	Rapeseed, soybean, and maize
22	Africa	Malawi	< 0.1	Cotton, cowpea, and banana
23	Europe	Portugal	< 0.1	Maize
24	Asia	Indonesia	< 0.1	Cotton
25	Asia	Bangladesh	< 0.1	Eggplant
26	Africa	Nigeria	< 0.1	Cowpea
27	Africa	Eswatini	< 0.1	Cotton
28	Africa	Ethiopia	< 0.1	Cotton
29	North America	Costa Rica	< 0.1	Soybean and cotton
		Total	190.4	

In 2019, the largest area of GM crops was possessed by soybean 48%, GM maize occupied an area of 60.9 million hectares globally, around 32% of the global maize production (**Figure 3**) (Turnbull et al., 2021). GM cotton covered 14% of the global area of cotton production in 2019 with 25.7 Mha of area. While GM canola occupied 5% from its 27% of global production in 2019 (Turnbull et al., 2021). In contrast to GM maize, soybean, canola and cotton, some of the GM crops that were planted in different countries also included sugarcane, papaya, alfalfa, squash, apples and sugar beets.

There has been a sharp increase in the approval of the number of plant species with GM varieties. Around 44 countries have provided regulatory acceptance to 40 GM crops and to 509 events of genetic modification since January 2022 (ISAAA, 2020). This manipulation includes 41 commercial traits for use in cultivation, food, and feed.

## 3 Concerns and related issues of GM crops production

The inception of GM crops has been controversial mainly due to the ethical concerns and issues of sustainability surrounding the negative impacts of GM crops. These issues range in different forms such as the detrimental effects of GM crops on the environment and human health, the ideology of creating new life forms within the society, and the intellectual property ownership of GM crops that provides economic benefits to specific people (Oliver, 2014). Most of these issues arise due to the arguments that farmers and seed companies attain the benefits of the GM crops rather than the consumer (Raman, 2017).

### 3.1 In relation to the environment

**Figure 4:**
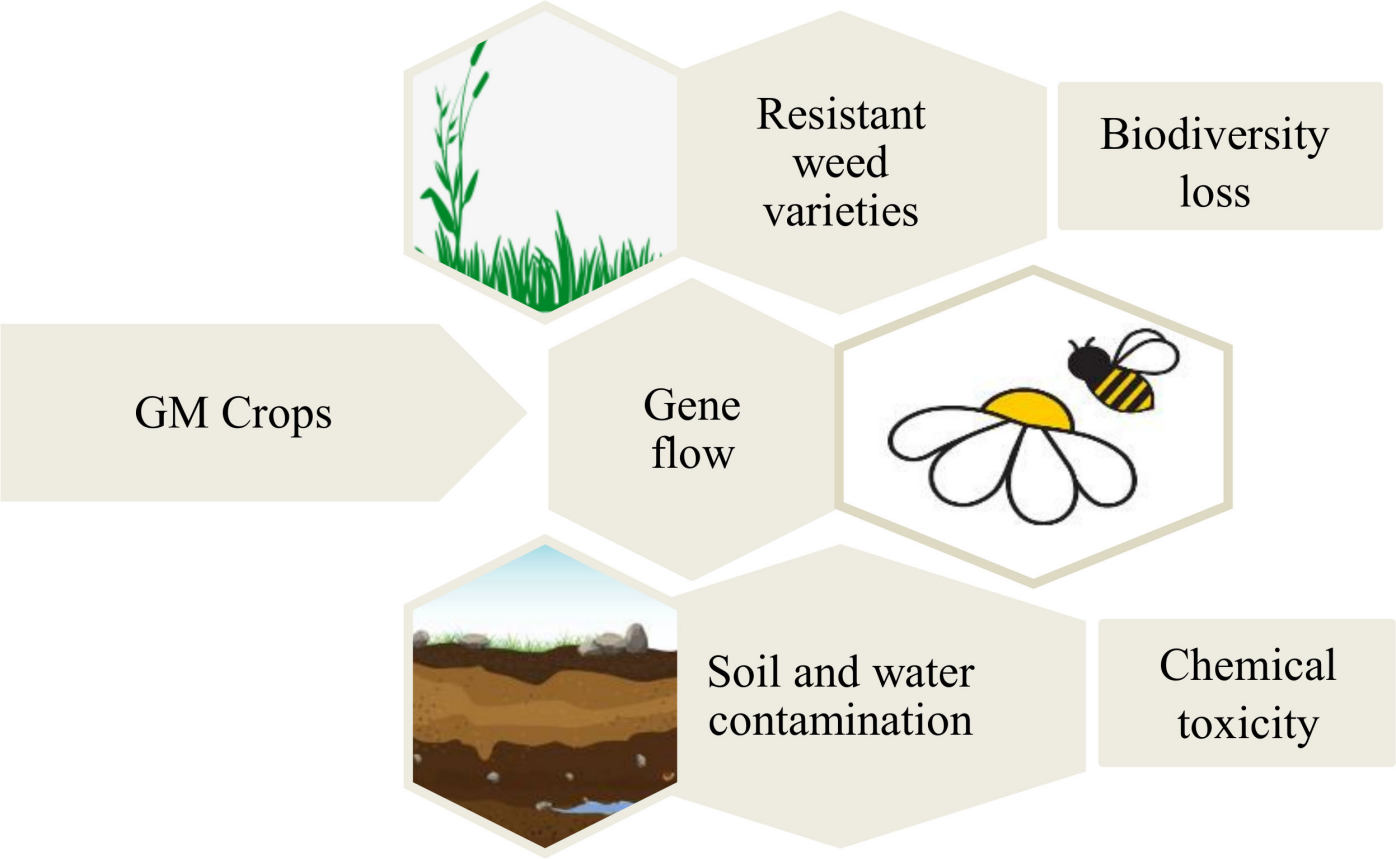
Major environmental concern related to the GM crops. The manipulated crops are widely prevented for their gene flow and its detrimental effect on the natural resources and biodiversity.

The introduction of GM crops may cause adverse impacts on the environmental conditions, which has been raised ethically by certain sections of the society (**Figure 4**). It has been argued that the GM crops pose a threat to the decline of crop biodiversity due to of the hybridization of GM crops with related non-GM crops through the transfer of pollen (Fernandes et al., 2022). The GM crops may become invasive over time and affect the population of local wild crop species. The use of specific chemical herbicides for controlling weeds that grow in the fields with GM crops tolerant to that chemical herbicide will lead to the appearance of highly resistant weeds that will be difficult to control. Due to the high use of chemicals to control those weeds, soil and water degradation can also occur (Sharma et al., 2022). The use of GM crops can have negative impacts on non-target organisms such as predators and honeybees (Roberts et al., 2020). For instance, the spread of the genetically manipulated herbicide tolerant corn and soybean, with the use of chemical herbicides has damaged the habitat and population of the monarch butterfly in North America (Boyle et al., 2019). It is considered that such environmental risk raised by the GM crops are difficult to be eliminated.

### 3.2 In relation to the human health

**Figure 5:**
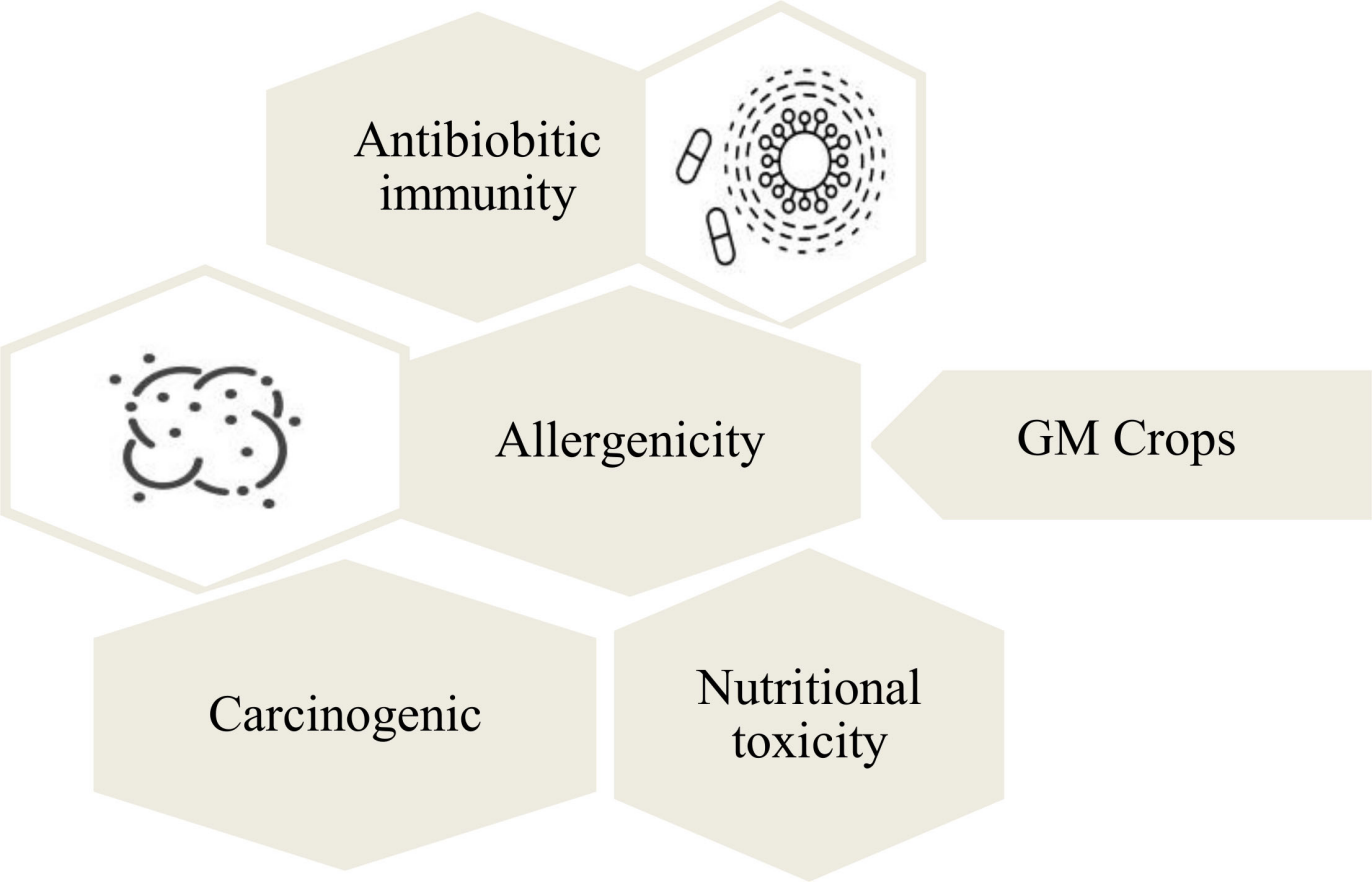
Human health related threats of GM crops. The consumption of GM crops is widely associated with toxicity and allergenicity of human beings.

The biggest ethical concern for the genetic modification of crops is their harmful effects on the human beings (**Figure 5**). It is assumed that consumption of the GM crops can result in the development of certain diseases that can be immune to antibiotics (Midtvedt, 2014). This immunity develops through the transfer of antibiotic resistant gene from the GM crops into humans after the consumption (Midtvedt, 2014). The long-term effects of GM crops are not known, which decreases their consumption rate. It is also found that a number of cultural and religious communities are against these crops and considers them detrimental for humans. It is believed that GM crops can trigger allergic reactions in human beings. In a study conducted for enhancing nutritional quality of soybeans (Glycine max), a methionine-rich 2S albumin from the Brazilian nut was transferred into transgenic soybeans. Since the Brazil nut is a common allergenic food, the allergenicity testing of transgenic soybean indicated allergenic reaction on three subjects through skin-prick testing. This allergenicity was associated to the introduction of 2S albumin gene of Brazil nut into the soybeans (Nordlee et al., 1996; EFSA et al., 2022). There are also assumptions that GM crops can cause the development of cancerous cells in human beings (Touyz, 2013). It is argued that cancer diseases are caused due to the mutations in the DNA, and the introduction of new genes into human body may cause such mutations (Mathers, 2007). Antibiotic resistance genes from genetically modified plants, used as selectable marker genes can get transferred to bacteria in the gastro-intestinal tract of humans (Karalis et al., 2020). However, the risk of such occurrence is very low, but it has to be considered when assessing the biosafety of the transgenic plants during field trials or commercialization approvals. The health risk of foods derived from genetically engineered crops are still being debated for rigorous evidence among the scientific community.

### 3.3 In relation to the development and intellectual property rights of GM crops

In the ethical debate of GM crops for sustainability, the philosophical reasons are fundamental against the development of these crops. It is viewed that genetic modifications of crops are inappropriate interference in the life of an organism (Evanega et al., 2022). The gap in this ethical ideology is aggravated in developing countries due to the prominent role of large Biotech companies in deciding how life forms are to be altered to make benefit from them. The concerns of the intellectual property rights, patents of these crops and their ownerships are at the heart of the ethical issues (Xiao and Kerr, 2022).

The private sector provides the majority of agricultural inputs such as the fertilizers, pesticides and seeds of improved crop varieties that farmers stored and reused season to season (Lencucha et al., 2020). This practice of seed reuse has made it difficult to gain benefits from the investments in artificial breeding. Nonetheless, production of hybrid species and advances in genetic technologies, it became possible to protect the new crop varieties that were developed, especially the larger-volume crops, such as the soybean and maize plants (Liu and Cao, 2014). This is particularly true for the genetic modification tools, which provide producers a stronger intellectual property right for their plants (Brookes and Barfoot, 2020). The patent rights provide monopoly power to the seed companies, which require the farmers to purchase the seeds from the patent owners during each year of plantation (Maghari and Ardekani, 2011). These seeds are known as terminator seeds that develop into infertile crops. The terminator technology was used for developing such seeds that prevented the diversion of genetic modifications to other plants, but limited farmers seed propagation (Niiler, 1999). This made farmers to purchase new seeds during each growing season, giving seed producers larger authority over the utilization of their seeds. It is considered to be ethically wrong to develop plants whose seeds are sterile that farmers cannot use for the second year of plantation (Bawa and Anilakumar, 2013). However, terminator seeds that produced infertile crops were temporarily terminated. The intellectual property rights for the GM crops provided protection to the crop varieties and limited farmers in using the seeds of GM crops for another cycle (Rodrigues et al., 2011). Moreover, intellectual property rights created a barrier for innovation as it provided a limited access to GM crops for several purposes (Redden, 2021).

Despite of these concerns of the GM crops, they are considered as one of the tools for achieving the sustainable food production. However, it needs to be evaluated for possible solutions for their negative impacts in securing their benefits.

## 4 Potential solutions for growing, commercializing, and incorporating GM crops into sustainable food production systems

**Figure 6:**
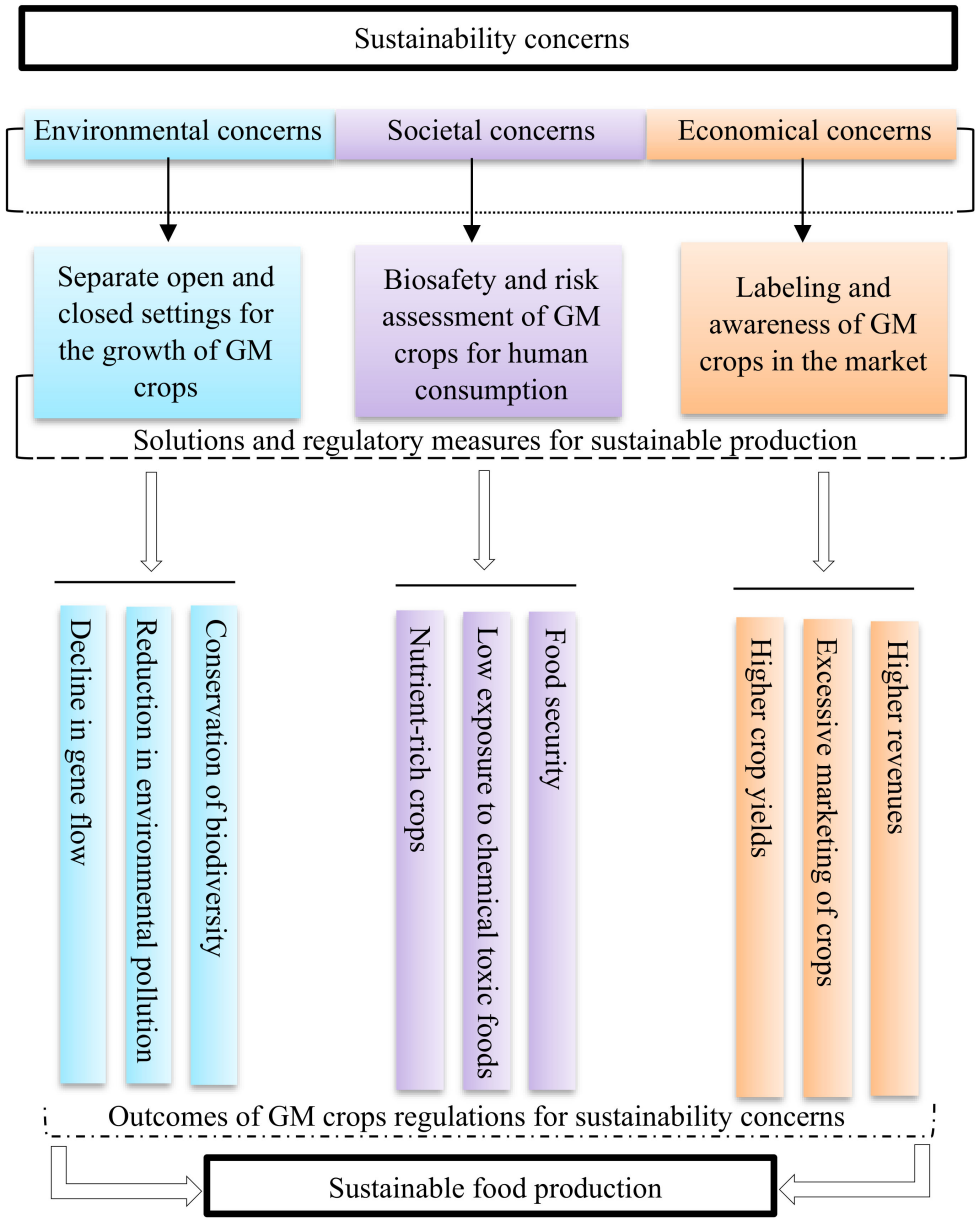
Schematic representation of the pathway for countering the concerns of GM crops. The development of separate settings, regulatory framework for biosafety and risk assessments, and commercialization continuum for GM crops will lead to their beneficial impacts, which will result in meeting the goals of sustainability.

The detrimental effects of the GM crops can be reduced or eliminated through appropriate measures that needs to be taken at different stages of incorporation, marketing and human consumption for ensuring that the GM plants are as harmless as the non-GM crops. This will lead to meeting the goals of sustainability and allow for the incorporation of GM crops into sustainable food production (**Figure 6**).

### 4.1 Towards the negative impacts on the environment

One of the major concerns of GM crops is their potential damage to the environment. It affects the environment through the gene flow that occurs from the GM crops to the neighboring non-GM crops *via* the pollen, a phenomenon known as the genetic pollution (Fitzpatrick and Ried, 2019). It is stated that genetic pollution will result in decline of biodiversity. However, the transfer of genes can occur through the pollen of plants at a distance between 50 m to 100 m (Carrière et al., 2021). Therefore, a feasible solution suggested towards the use of GM crops is the practice of growing the GM crops at a distance farther away from the non-GM crops that will lower the chances of gene flow. In addition, such a solution will contribute towards the lowering of crop pollen viability and competitiveness after moving through long distance between the plants (Nishizawa et al., 2010). It was reported in a study that a gene resistant to herbicide from a field of genetically modified oilseed rape moved to the neighboring non-genetically modified oilseed rape (Nishizawa et al., 2010). The investigation from this study indicated that one out of ten thousand oilseed rape contained the modified gene at a distance of 50 m (Nishizawa et al., 2010). Therefore, it is suggested to grow the GM crops at a distance of 50 m away from the non-GM crops during their use in sustainable agriculture, as this practice will reduce the percentage of gene flow (Carrière et al., 2021).

### 4.2 Towards the negative impacts on the societal and community health

The sustainable agriculture is focused on the health effects of GM crops on the current and future generations. The health effects of GM crops remain an ethical issue that needs to be investigated due to lack of direct studies on the human health effects and the consumption of GM crops (Garcia-Alonso et al., 2022). The possible solutions towards the health effects of GM crops are the constant regulation of these crops through different biosafety testing and risk assessment by health authorities before consumption (Akinbo et al., 2021). The biosafety testing of GM crops should consider the standard that foods developed from GM plants are intended to be as safe as genetically similar varieties of non-GM plants. To date there is no solid evidence that GM crops approved in the US and other countries have harmed humans or animals that had consumed them. This highlights that the safety assessment of GM crops is quite robust. However, to predict the adverse effect of GM crops consumption on human health, scientifically sound and long-term studies need to be conducted under controlled and validated experimental conditions on animals such as rats, cows, pigs, etc.

### 4.3 Towards the negative impacts on the economy

Since the GM crops are passing through various regulatory measures and meeting the testing standards, these crops are still prevented from release to the market (Davison and Ammann, 2017). For instance, with the introduction of new drugs, people are always given a choice to be the first users or second users, but after certain stages of testing, the drugs are released to the market for the use of everyone, in that ground it would be unethical to prevent the release of GM crops after testing and meeting the regulatory measures (Teferra, 2021). The holding of the release of GM crops to the market prevents the economic benefits that countries can attain through their production. However, a solution has been developed towards such issue is by labelling of the GM crops for the market sales (Delgado-Zegarra et al., 2022). Such labelling’s allow for the consumer’s sovereignty as the people have the fundamental right to know what food they are consuming and about the processes involved in its production (Yeh et al., 2019). Positive information about GM crops needs to be brought into the public in comparison to the negative assumptions for improving their marketability. The surveys conducted on the public opinions in a study indicated that majority of the people in USA supports labelling of GM crops (Wunderlich and Gatto, 2015). According to the Food and Drug Administration (FDA), the labelling of GM crops is not to indicate if they are harmful, but rather to describe the attributes of these crops to the public (Borges et al., 2018).

## 5 Genome editing in the new era as a promising solution for crop manipulation

The scientists have developed advanced molecular tools for the precise modification of plants. Zinc finger nucleases (ZFN) was developed in 2005 with *Nicotiana tabacum* plants for plant trait improvements (Raza et al., 2022). A ZFN is a synthetic endonuclease that is composed of a designed zinc finger protein (ZFP) joined to the cleavage domain of a restriction enzyme (FokI) (Paschon et al., 2019). It can be redesigned to cleave new targets by creating ZFPs with new selected sequences. The process of cleavage event instigated by the ZFN causes cellular repair processes that in turn mediate efficacious manipulation of the desired locus. Within a passage of five years, transcription activator-like nucleases (TALENS) were developed as a new genome editing technique (Raza et al., 2022). Transcription activator-like effector nucleases (TALENs) introduces specific DNA double-strand breaks (DSBs), as an alternative method to ZFNs for genome editing (Forner et al., 2022). TALENs are identical to ZFNs and consists of a non-specific domain of FokI nuclease fused to a changeable DNA-binding domain. This DNA-binding domain possesses highly conserved repeats acquired from transcription activator-like effectors (TALEs) (Tsuboi et al., 2022). These are proteins synthesized by the bacteria Xanthomonas to prevent the transcription of genes in host plant cells.

Although these two techniques have modernized plant genomics, each had its own limitations. However, in 2013 emerged the new editing technique named CRISPR/Cas9 (clustered regularly interspaced short palindromic repeats), which provided the plant breeders a widespread ability to make targeted sequence variations, resulting in rapid improvement of crops (Nekrasov et al., 2013). This technique of genome editing uses site-directed nucleases (SDN) to make exceptionally precise incisions at a particular region of DNA (Metje-Sprink et al., 2019; He and Zhao, 2020). SDN techniques are classified into three categories: SDN-1, SDN-2, and SDN-3 (Lusser et al., 2012). The SDN-1 technique instigates a single or double stranded break to remove a part of the DNA, while SDN-2 technique utilizes a small donor DNA template to induce a desired mutation sequence. The third technique, SDN-3 uses a much longer donor DNA template that is introduced into the target DNA region, which makes this technique similar to traditional recombinant DNA technology (Podevin et al., 2013).

### 5.1 CRISPR/Cas9 tool for plant genome editing

The CRISPR/Cas system is composed of CRISPR repeat-spacer arrays and Cas proteins. It is a bacterial RNA mediated adaptive immune system that safeguards against bacteriophages and other harmful genetic components by breaking the foreign nucleic acid genome (Hu and Li, 2022). The CRISPR system is based on the RNA-guided interference (RNAi) with DNA (Koonin et al., 2017). This system is divided into two classes based on their Cas genes and the type of the interference complex. Class 1 CRISPR/Cas systems utilize multi-complexes of Cas proteins for interference, while class 2 systems use construct having single effector polypeptides with CRISPR RNAs (crRNAs) to perform interference (Hu and Li, 2022).

**Figure 7:**
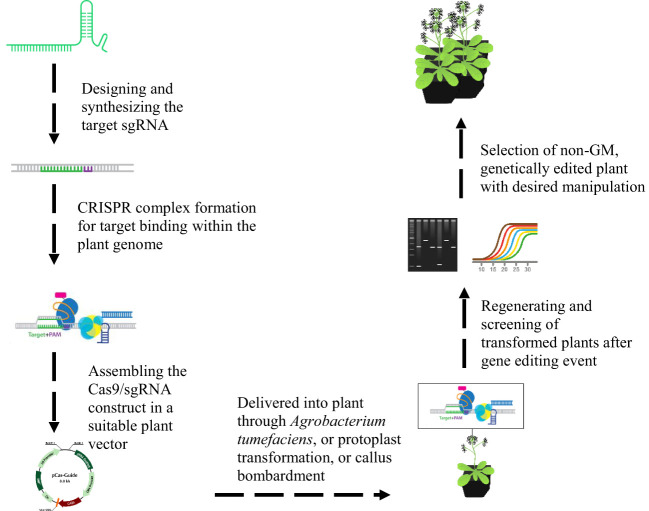
Illustration of CRISPR CAS9/sgRNA plant genome editing system. The designing of sgRNA is performed using the available online resources for the target gene. The CRISPR complex is formed with target sgRNA and suitable Cas9 variant, which will be cloned into a plant vector for the target plant species transformation with a suitable technique of transformation. The putative transformed plants will be selected after identifying the Cas9 and target sgRNA based on the screening through PCR or RE genotyping and DNA sequencing. The plants with edited genome will be selected and regenerated.

In comparison to TALENS and ZFN, CRISPR system can target multiple sites using several single guide RNA’s (SgRNAs) with a single Cas9 protein expression (**Figure 7**). This kind of multiplex editing has sophisticated its use in genome engineering and pyramid breeding (Chen et al., 2019). It can create multigene knockouts and knock-ins, chromosomal translocations and deletions (Salsman and Dellaire, 2016). Various approaches have been employed for multiplex guide RNA (gRNA) expression with one cassette in plants. The editing efficiency can be maintained with one promoter to attain consistent expression of each gRNA by placing it into a small vector (Chen et al., 2019). This has been attained by utilizing a polycistronic gene, having interspersion of gRNA within Csy4 recognition sites, transfer RNA sequences, and ribozyme sites, refined in the cell to produce mature gRNAs for modification (Gao and Zhao, 2013; Xie et al., 2015; Cermák et al., 2017). Moreover, the potential of the discovered new generation of CRISPR nuclease termed as Cpf1 that initiates its own crRNA, has been an efficient system for complex genome editing in crops (Wang et al., 2017).

### 5.2 CRISPR/Cas9 application for SDGs

In the year 2015, the SDGs were launched. It consisted of 17 SDGs, with enhanced human health, poverty eradication and improved food security being the three important goals (Aftab et al., 2020). All the SDGs have been set for an achievement date of 2030. The successful achievement of these essential and valuable goals requires substantial adoption of technology and innovation. Advancements in plant breeding have resulted in efficient food production systems since the middle 20th century (Smyth, 2022). With further improvement in the current era of agricultural biotechnology through CRISPR/Cas9 system, crop yield improvements, nutritional enhancements, and reduced environmental impacts are possible (Tripathi et al., 2022). This indicates the potential role of genome editing technologies and highlight their important role in achieving the three essential SDGs. CRISPR/Cas9 technique enhances the sustainability and improves global food security in various ways.

#### 5.2.1 Abiotic stress tolerance

Under abiotic stresses, CRISPR/Cas9 based genome editing is a propitious tool for developing resilient cultivars for sustainable agricultural production. Drought, salinity, and temperature stresses have been significant abiotic stresses studied using CRISPR/Cas9 (Wang et al., 2020). In tomato, two drought stress responsive genes, such the non-expressor of pathogenesis-related gene 1 (*SlNPR1*) and mitogen-activated protein kinase 3 (*SlMAPK3*), were knocked out by CRISPR/Cas9 system, with no improvement in drought tolerance observed (Wang et al., 2017b). Salt tolerant alleles were identified in functional analyses of genes related to the reception of salt stress. In tomato, blocking the activity of Salt Overly Sensitive 1 (*SOS1*) gene, which is a Na^+^/H^+^ antiporter for controlling Na+ levels in root epidermal cells, resulted in reduced salt tolerance (Wang et al., 2020). Moreover, the precise manipulation of protein domains of tomato hybrid proline-rich protein1 (HyPRP1), a negative regulator of salt stress responses, provided enhanced salinity tolerance to the edited tomato plants (Tran et al., 2021).

Climatic changes accompanied with large variations in temperature affects cropping. The function of genes in temperature stress reactions are essential for developing and breeding temperature tolerating crops. In relation to this, CRISPR/Cas9 system was used to knockout a chilling related gene and a heat responsive factor, tomato C-repeat binding factor 1 (*SlCBF1*) and Brassinazole Resistant 1 (*SlBZR1*). It was shown that these genes were firmly associated with temperature tolerance as the altered alleles of *SlCBF1and SlBZR1* displayed lessened chilling and heat stress tolerance, respectively (Yin et al., 2018).

In another study, CRISPR/Cas9 triple knockout of *pyl1*, *pyl4*, and *pyl6*, *OsPYL* abscisic acid receptor gene family displayed high-temperature tolerance, increased grain yield, and lower preharvest sprouting in comparison to wild type (Miao et al., 2018). Genome-edited crops contribute towards improved water and nitrogen use efficiency as demonstrated through field trial and experimental data (Wen et al., 2021). Research on enhanced drought tolerance of cotton developed through genome editing tools demonstrated environmental footprint of cotton production (Peng et al., 2021). Arable lands are also heavily contaminated with metal toxicity. Rice varieties with reduced level of arsenic, radioactive cesium, and cadmium were obtained *via* CRISPR/Cas9 knockout of *OsHAK1*, *OsARM1*, and *OsNramp5* (Nieves-Cordones et al., 2017; Tang et al., 2017; Wang et al., 2017a).

This application of genome editing can aid towards the SGD-13 and SDG-15, which are to promote more environmentally sustainable agriculture. The building of sustainable environment improves the life of organisms on earth. The progression in enhancing the sustainability of present agricultural systems is vital due to the pressures of climatic changes, clearing of forest lands and utilization of arable lands for non-agricultural activities. Without research focuses on genome editing, decline in yields due to the impacts of climatic changes can severely damage food security. Hence, abiotic stress tolerance can also help towards SDG-2 for reducing hunger through food production under various climatic conditions.

#### 5.2.2 Biotic stress tolerance

Crop yields and quality are largely affected by biotic stress factors. Several plants are made resistance to insects, bacterial, viral and fungal diseases through CRISPR/Cas9 knockout. For instance, with CRISPR/Cas9, wheat varieties resistance to one of common fungal disease, powdery mildew has been created *via* knocking out of all six TaMLO alleles responsible for powdery mildew (Wang et al., 2014). Furthermore, CRISPR/Cas9 knockout of *OsERF922*, an ethylene responsive gene, generated blast-resistant rice, resistant to a devastating fungal rice disease (Wang et al., 2016). Crops are also affected by bacterial blight generated by *Xanthomonas oryzae*. In rice plants, excision of OsSWEET13 promoter resulted in the development of blight resistance plants (Zhou et al., 2015). In relation to viral diseases, CRISPR/Cas9 technique has produced several resistance plants such as tungro disease–resistant rice (Macovei et al., 2018), cotton leaf curl disease–resistant (Iqbal et al., 2016), and broad potyvirus–resistant cucumber (Chandrasekaran et al., 2016). Lately, Lu et al. (2018a) demonstrated that altering *OsCYP71A1* gene resulted in serotonin biosynthesis blockage that heavily increased the level of salicylic acid, resulting in resistance to two destructive plant pests, plant hoppers and stem borers.

*Pseudomonas syringae* is the causual agent of bacterial speck disease, a major threat to tomato productions (Cai et al., 2011). In an early application, CRISPR/Cas9 was utilized to knockout a positive regulator of downy mildew disease in tomato, which generated tomato mutant alleles ortholog of downy mildew disease resistance in Arabidopsis 6 (DMR6). It was found that the mutant lines showed resistance against *P. syringae*, spp.*Xanthomonas* spp. and *Phytophthora capsica* (Paula et al., 2016). The mutant lines were highly useful resources for breeding tomato plants. In another common biotic stress, susceptibility to *Oidium neolycopersici* infection was associated to few members of the transmembrane protein Mildew Locus O (MLO). It was identified that among the 16 MLOs in tomato, the profound gene was *SlMLO1*, and its innate mutants with loss-of function displayed resistance towards powdery mildew disease (Zheng et al., 2016). The mutant strains generated *via* CRISPR/Cas9 containing homozygous SlMLO1 alleles, 48-bp truncated versions of the wild SlMLO1, exhibited resistance towards *O. neolycopersici* infection. Similarly, Nekrasov et al. demonstrated that CRISPR/Cas9 derived knockout of MLO provided powdery mildew resistance to tomatoes (Nekrasov et al., 2017). It was also found that the *SlMLO1* plants produced through CRISPR/Cas9 technique were devoid of any foreign T-DNA sequence, which made them indistinguishable from natural *SlMLO1* mutant plants (Nekrasov et al., 2017).

In addition to major bacterial, viral, and fungal diseases, CRISPR/Cas9 was applied for other biotic stresses of oomycete infections. In papaya, *Phytophthora palmivora* is a devastating agent of oomycete disease. A papaya mutant plant was developed with a functional cysteine protease prohibitor (*PpalEPIC8*) that resulted in enhanced *P. palmivora* resistance (Gumtow et al., 2018). Similarly, cocoa beans have been made resistance towards another oomycete pathogen, *Phytophthora tropicalis*, *via* the CRISPR/Cas9 system (Fister et al., 2018).

Similar to abiotic stress tolerance generating biotic stress tolerance can also lead to SDG-15, as it will lead enhanced living condition for the plants. This will also result in creation of a sound environment for different organisms that depend on plants for their survival and growth.

#### 5.2.3 Crop yield enhancement

The genome editing tools are employed primarily for improving crop yield which is a composite characteristic that relies on various components. CRISPR/Cas has been used to knock-out negative regulators that influences yield controlling factors such as grain weight (*TaGW2*, *OsGW5*, *OsGLW2*, or *TaGASR7*), grain number (*OsGn1a*), panicle size (*OsDEP1*, *TaDEP1*), and tiller number (*OsAAP3*) for achieving the contemplated traits in plants with loss-of-function alteration in these genes (Li et al., 2016; Li et al., 2016; Zhang et al., 2016; Liu et al., 2017; Zhang et al., 2018a; Lu et al., 2018b). In rice, using CRISPR system, simultaneous knockout of various grain weight related genes (*GW2*, *GW5*, and *TGW6*) led to trait pyramiding that efficiently increased grain weight (Xu et al., 2016). Huang et al. (2018) recently combined CRISPR/Cas9 with pedigree analysis and whole-genome sequencing for the large identification of genes that were responsible for composite quantitative traits, including yield. The study analyzed 30 cultivars of the Green Revolution miracle rice cultivar (IR8) and identified 57 different genes in all high-yielding lines, to be used for gene editing *via* Cas9 knockout or knockdown system. Phenotypic trait analysis indicated the role of most of these genes in determining yield of rice. It laid insight on yield improvement and facilitated the molecular breeding of improved rice varieties.

A high yielding commercial corn was produced by DuPont Pioneer through the CRISPR/Cas9 knockout in waxy corn line (Waltz, 2016a). Genome editing techniques are also used to develop semi-dwarf corn varieties having higher production and low height, in order to lower moisture and nutrient requirements of the corn (Bage et al., 2020). Moreover, in maize, multiple grain yield traits were enhanced by creating fragile promoter alleles of *CLE* genes, and a null allele of a recently spotted partially redundant recompensing *CLE* gene, utilizing CRISPR/Cas9 technique. Considerable gene editing research is being undertaken on wheat for increased yield, seed sizes, and seed weight (Li et al., 2021b). Although the future of plant genome editing remains uncertain in Europe, researchers of Vlaams Instituut voor Biotechnologie (VIB) of Belgium have currently applied to undertake three genetically edited corn varieties for field trials that have higher yields and enhanced digestibility (VIB, 2022).

Most of the genome edited crops for yield enhancement will lead to increased farm and household revenues. This results in reducing poverty. Although few studies are conducted to date on this discrete goal measurement. It has been reported in one study that the acquisition of Bt cotton developed through transgenesis approach in India, has increased the income by 134% for farmers living with less than 2 USD/day (Subramanian and Qaim, 2010). This was mainly due to improved yields and lowered inputs costs. The potential of genome editing for yield increases indicates that, similar to GM crop adoption, genome edited crops can also improve the incomes of the farmers. The early evidence related to possible increase in the household income due to yield increases, indicates that genome editing makes significant contributions to SDG-1for eradicating poverty. The significant genome editing studies for increasing the yield of major food staple crops and other essential crops indicate the substantial potential of GMs in contributing towards SDG-2, which aims to end hunger and achieve food security.

#### 5.2.4 Quality improvement

The quality of crops may differ depending on the various breeding techniques used. The genome editing has impacted several quality traits such as nutrition, fragrance, starch content and storage quality of crops. Using CRISPR/Cas9, the knockout of Waxy, resulted in enhanced rice eating and cooking quality with low amylose content (Zhang et al., 2018b). Resistant starch rich varieties with elevated amylose were developed by altering the starch connecting enzyme gene, *SBEIIb*, by CRISPR/Cas9. Consuming food with increased amylose content is essential for the patients with diet-related to noninfectious chronic diseases (Sun et al., 2017). Another important quality for the commercial and edible rice varieties is the fragrance. The biosynthesis of a major rice fragrant compound, 2-acetyl-1-pyrroline is due to a variation in the betaine aldehyde dehydrogenase 2 (*BADH2*) gene. With TALEN genome editing tool, specific alteration of *OsBADH2* resulted in a fragrant rice variety with low 2-acetyl-1-pyrroline content identical to the innate fragrant rice variant (Shan et al., 2015).

In Western countries, celiac disease is triggered due to cereal crops Gluten protein in more than 7% of individuals . Wheat plant consists of nearly 100 genes or pseudogenes, α-gliadin gene family, for gluten-encoding. CRISPR/Cas9 system allows for newer pathways to modify traits governed by massive gene families with unessential properties. At present, researchers have created low-gluten wheat by simultaneous knockout of most conserved domains of α-gliadin family (Sanchez-Leon et al., 2018). Furthermore, other high-quality plants produced by CRISPR/Cas9 includes *Camelina sativa* (Jiang et al., 2017) and *Brassica napus* (Okuzaki et al., 2018) plants with high oleic acid oil seeds, long shelf-life varieties of tomato (Li et al., 2018a), enhanced lycopene tomatoes (Li et al., 2018) or γ-aminobutyric acid content in tomatoes (Li et al., 2018b), and resulted in low levels of toxic steroidal glycoalkaloids in potatoes (Nakayasu et al., 2018). The increased lycopene production acts as an antioxidant for lowering the risk of cancer and heart diseases (Zaraska, 2022). Recently in UK, Rothamsted Research has received acceptance for field trials of genetically edited wheat that synthesizes lower asparagine, a potential cancer producing compound in the toasted breads (Case, 2021).

Genome editing applications surrounding the quality improvement has the prospective to make considerable contributions to SDG-3. Quality improvements in crops promote human health and well-being. Moreover, the capability of genome editing in producing food that may avert specific diseases are directly associated with beneficial health implications.

#### 5.2.5 Nutritional enhancement

One of the applications of genome editing is to enhance the nutritional metabolism and decrease the undesirable substances from the crops through gene expression regulation. In 2021, Japan launched the first genome-edited tomato Sicilian Rouge High GABA (gamma-aminobutyric acid). The edited variety has around four to five times higher amount of GABA than the ordinary tomatoes. The increase was the result of CRISPR/Cas9 genome editing that targeted the autoinhibitory domain (AID) of GAD3 on the C-terminal side, an enzyme involved for the GABA biosynthesis (Nonaka et al., 2017). A frameshift mutation was induced in this autoinhibitory domain that caused the early termination of translation, and the excision of autoinhibitory domain of GAD3 (Nonaka et al., 2017). This strategy eliminated the inhibitors of GAD3 and increased the enzymatic activity involved in the GABA biosynthesis, whose activity is generally suppressed without manipulating the expression level of GAD3. Furthermore, CRISPR/Cas9 system was also utilized to improve the total wheat protein content with enhanced grain weight with the knockout of *GW2* gene that encodes for a RING-type E3 ubiquitin ligase, known to govern the cell numbers of spikelet hulls (Zhang et al., 2018a). Moreover, genome editing was applied to lettuce that has produced a new variant synthesizing enhanced levels of thiamine, β-carotene, and vitamin C (Southy, 2022).

Research is additionally focused on enhancing corn vitamin A content and provitamin A (Maqbool et al., 2018; Xiao et al., 2020). In the US, a genome editing study was targeted on increasing wheat fiber content. The research is underway for the field trials of this new enhanced fiber wheat (Knisley, 2021). Ensuring sufficient nutrient content in human diets enhances life-long health benefits and prevents the debilitating diseases. The genome editing tools promising results in broad applications of nutritional enhancements is essential for food insecure developing countries. With this application, the genetic editing underpins the other portions of SDG-2 and SDG-3, which are to achieve and consume fortified nutritional food.

#### 5.2.6 Enhancing hybrid breeding

Hybrid breeding is an appropriate method for enhancing crop productivity. A male-sterile maternal line is essential for producing an improved-quality hybrid variety. Through CRISPR/Cas9 technique, tremendous progress has been made to produce male-sterile lines, which includes photosensitive genic male-sterile rice (Li et al., 2016), heat sensitive male-sterile lines in rice (Zhou et al., 2016), wheat (Singh et al., 2018), and corn (Li et al., 2017a). Heterosis in breeding faces hybrid sterility as an obstacle. Reproductive barriers were disrupted in hybrids between japonica and indica, SaF/SaM (sterility locus Sa) (Xie et al., 2017a) and African rice (Oryza glaberrima Steud) OgTPR1 (sterility at the S1 locus) (Xie et al., 2017b). It was found that knockout of the indica *Sc* gene in the allele Sc-I protected the male fertility in japonica-indica hybrids (Shen et al., 2017). Likewise, it was shown that knockout of the toxin gene *ORF2*, improved the fertility of japonica-indica hybrids (Yu et al., 2018). Furthermore, in rice plants, genetic editing was utilized to replace mitosis for meiosis, through the knockout of three important meiotic genes, PAIR1, OSD1, and REC8 (Mieulet et al., 2016). Moreover, simultaneous activation of BBM1 in egg cells or knockout of MTL, by two independent research groups resulted in asexual propagation lines that fixed the hybrid heterozygosity through seed propagation (Khanday et al., 2018; Wang et al., 2019a). In addition, gene editing is also a constructive method for enhancing haploid breeding (Yao et al., 2018), shortening growth periods (Li et al., 2017b), improving resistance to silique shatter (Braatz et al., 2017), and countering the self-incompatibility of diploid potatoes (Ye et al., 2018), that meets the requirements of breeders. The enhanced breeding of hybrid plants results in the developing of novel plant varieties that supports the SDG-15, enhancing life on land through diverse plant species. Therefore, the successful application of genome editing technologies have modified and improved many essential traits in diverse crops for the achievement of different SDGs (**Table 2**).

**Table 2:** Overview of the recent CRISPR/Cas9 applications for the SDGs.

Applications	Plants	Target genes	Traits	SDGs	References
Abiotic and Biotic stresses tolerance	Arabidopsis	HSFA6a and HSFA6b	ABA and osmotic tolerance	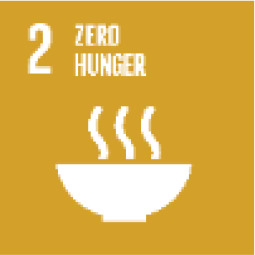 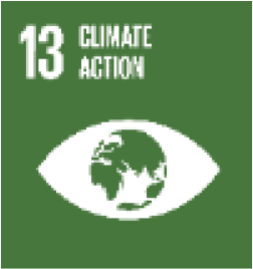 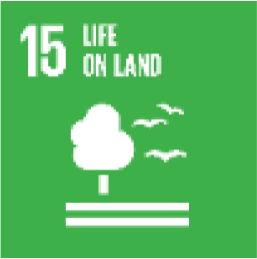	(Wenjing et al., 2020)
	Arabidopsis	AITR	Drought tolerance		(Chen et al., 2021)
	Rice	OsSAP	Drought tolerance		(Park et al., 2022)
	Rice	OsbHLH024	Salinity tolerance		(Alam et al., 2022)
	Rice	OsERA1	Drought stress		(Ogata et al., 2020)
	Rice	OsSWEET14	Bacterial blight resistance		(Zafar et al., 2020)
	Soybean	F3H1, F3H2, and FNSII-1	Mosaic virus resistance		(Zhang et al., 2020)
	Soybean	GmAITR	Salinity tolerance		(Wang et al., 2021)
	Chickpea	4CL and REV7	Drought tolerance		(Razzaq et al., 2022)
	Tomato	SlMAPK3	Heat tolerance		(Yu et al., 2019)
	Barley	HvARE1	Nitrogen use efficiency		(Karunarathne et al., 2022)
	Banana	DMR6	Downy mildew resistance		(Tripathi et al., 2021)
	Potato	StDND1 and StCHL1	Late blight resistance		(Kieu et al., 2021)
Enhanced yield	Rapeseed	BnaMAX1	Improved productivity	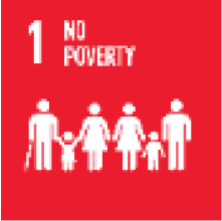 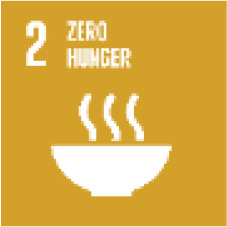	(Zheng et al., 2020)
	Wheat	GW7	Increase in grain size and weight		(Wang et al., 2019b)
	Rice	GS3	Improved productivity		(Huang et al., 2022)
	Soybean	GmFT2a and GmFT5a	Increased pod and seed size		(Cai et al., 2020)
Enhanced Quality	Rice	GW2	Enhanced protein	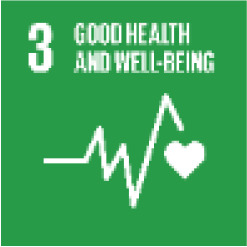	(Achary and Reddy, 2021)
	Rice	OsGAD3	Increased GABA		(Akama et al., 2020)
	Rice	GBSS	Low amylose		(Huang et al., 2020)
	Wheat	TaSBEIIa	High amylose		(Li J et al., 2021a)
	Wheat	CM3 and CM16	Reduced allergens		(Camerlengo et al., 2020)
	Wheat	TaASN2	Reduced asparagine		(Raffan et al., 2021)
	Potato	SBE1	Reduced asparagine		(Tuncel et al., 2019)
	Barley	D-hordein	Lowered prolamine and enhanced glutenin		(Yang et al., 2020)
	Rapeseed	BnITPK	Reduced phytic acid		(Sashidhar et al., 2020)
Hybrid breeding	Rice	Zep1	Improves the frequency of genetic recombination	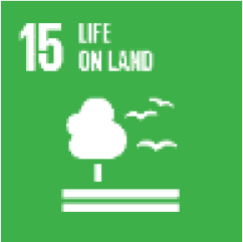	(Liu et al., 2021)
	Wheat	ZIP4-B2	Recombination of homologous chromosomes		(Martín et al., 2021)

### 5.3 Regulatory concerns of crop genome editing

The recent developments in biotechnology in the form of genome editing has made it viable for food products to get into the market quicker in a feasible rate. The latest genome editing tools are essential for the future production of crops. This is due to their robustness, process precision and timely regulation in comparison to conventional GM crops. Several products are now developed through CRISPR/Cas9 system that are not considered as GMO in several countries. It was stated by the US Department of Agriculture (USDA) that the crops edited *via* CRISPR/Cas9 platform can be grown and marketed without regulatory processes and risk assessments that are mandatory on GMOs biosafety regulations (Waltz, 2016b). Such a step will save millions of dollars spent on investigating GM crops through field tests and data collections, reduces the time required for introduction of improved crop varieties into the market, and removes the uncertainty associated with the consumption of GM crops within the public. To date, five crops developed through CRISPR/Cas9 system were accepted by the USDA without the regulatory measures of GMOs. These includes browning-resistant mushrooms, created through CRISPR/Cas9 technique by the knockout of polyphenol oxidase (*PPO*) gene (Waltz, 2016b). Likewise, waxy corn plants with enhanced amylopectin have been developed by CRISPR/Cas9 system with the inactivation of an endogenous waxy gene (*Wx1*) and introduced without regulations (Waltz, 2016a). *Setaria viridis* with delayed flowering period was attained through the deactivation of the *S. viridis* homolog of the corn *ID1* gene (Jaganathan et al., 2018), camelina altered for improved oil content (Waltz, 2018), and soybean with modified *Drb2a* and *Drb2b* for drought tolerant, were not subjected to GMO regulatory measures (Cai et al., 2015; Kumar et al., 2020).

## 6 Perspectives on the criticisms of GM crops incorporation into sustainable food production systems

**Figure 8:**
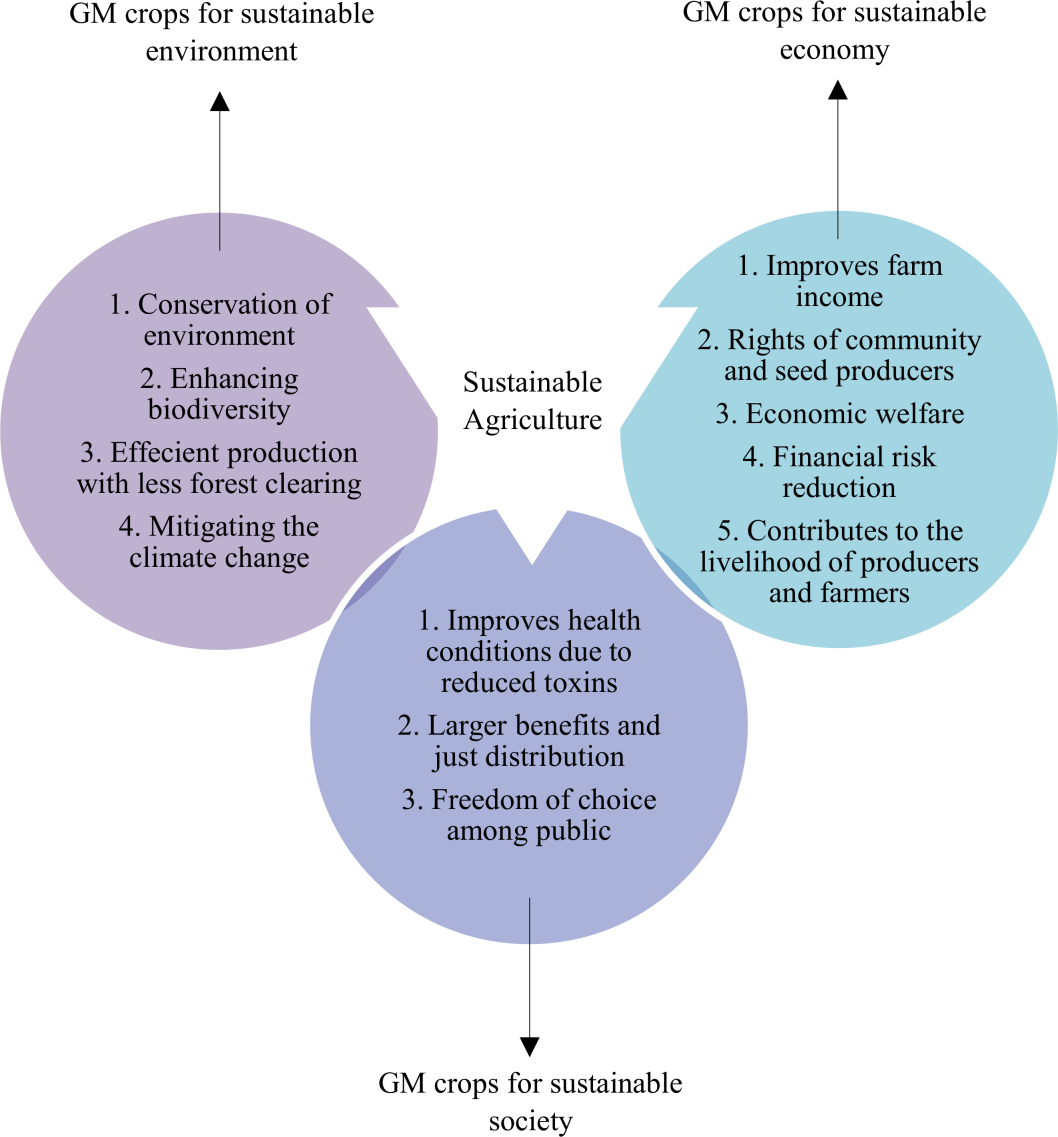
Production of GM crops operationalizes the three themes of sustainability, environment (efficient use of resources and preservation of biodiversity), society (freedom of choice and livelihood), and economy (national income improvement and financial risk reduction).

Agriculture plays an important role towards the SDGs achievement, such as for reducing hunger and malnutrition, alleviating poverty, implementing a sustainable production and consumption system, countering climatic changes, ensuring gender equality, improving energy use, and maintaining healthy ecosystem services (Viana et al., 2022). It acts as a basis for economic development in several countries. Global agriculture has successfully provided sufficient food for meeting the rising demand and varied consumption patterns of humans over the recent decades (da Costa et al., 2022). This has been possible largely due to the agricultural intensification at the expense of environmental resource degradation, biodiversity loss, harmful gas emissions, and land clearing (Liu et al., 2022). However, it has been shown that the advancement of the biotechnological tools for genetic modification of crops will allow agricultural practices to achieve SDGs in a sustainable manner. Nonetheless, GM crops faces the moral and ethical dilemma of their incorporation into the sustainable agricultural practices, which can be negotiated through the appropriate balance of benefits and negative impacts of GM crops by encompassing all the three relational aspects of sustainability, such as the environment, society, and the economy (**Figure 8**).

### 6.1 GM crops for sustainable environment

GM crops are scrutinized for the environmental safety. More than 300 million EUR were invested by the European Union (EU) in 130 research projects. It covered a research period of more than 25 years specifically to reach at the interpretation that GM crops are not riskier compared to conventional bred plants (European Commission, 2010). In fact, GM crops that were developed for input traits such as insect resistance and herbicide tolerance have resulted in a reduction to agriculture’s environmental footprint by enhancing sustainable farming practices (Brookes and Barfoot, 2015). Moreover, the genetic modification of crops is a logical continuation of selective plant breeding that humans have developed for thousands of years. It results in the conservation of environment and the plant biodiversity allowing their incorporation into sustainable food production systems. Klumper and Qaim undertook a meta-analysis of the initial data obtained from the farm surveys or field trials in various parts of the world (Klümper and Qaim, 2014). It indicated that the insect resistance of GM crops has lowered the pesticide application by 36.9%.

In addition, GM seeds contribute towards the adoption of conservation tillage, which are sown straight into the fields without early ploughing. This practice conserves the essential soil microorganisms, preserves soil moisture, and maintains carbon in the soil. A meta-analysis was performed by Abdalla et al. (2016) to compare the CO_2_ emissions of entire season from the tilled and untilled soils. It was found that on average, 21% more carbon was emitted from the tilled soils than the untilled soils. Furthermore, the use of powered agricultural machines was lowered due to less pesticide use and no/less field ploughing. This provides indirect benefits to sustainable agriculture by conserving fossil fuels and decreasing the emission of CO_2_ into the atmosphere. In the United States, the land area for the soybean production increased by approximately 5 million hectares between 1996 and 2009, and 65% of those fields were that of no-tillage practices field due to the adoption of GM soybeans (Brookes and Barfoot, 2016). This resulted in a decline of fuel utilization of 11.8%, from 28.7 to 25.3 liters per hectare, and an approximate reduction of greenhouse gas emissions of more than 2 Gt between 1996 and 2009. The genetically modified soybean fields presented similar impacts of reduction in greenhouse gas emission in various countries such as, Uruguay, Argentina and Paraguay (Brookes and Barfoot, 2016).

The cultivation of GM crops has also increased the biodiversity of non-target beneficial insects due to the lack of chemical use in the fields for the control of harmful insects (Karalis et al., 2020; Talakayala et al., 2020). The pest resistant traits of GM crops allow the restoration of crop species that were discontinued due to harmful insect pressure. In addition, it improved the crops adaption to several environmental conditions, allowing for a diversified production practice (Anderson et al., 2019). Despite the argument that GM crops threaten biodiversity, it is found that different practices of agriculture affect the biodiversity, and the GM crops do not broaden this threat.

Agriculture causes significant clearance of natural habitat for the food production (Mrówczyńska-Kamińska et al., 2021). However, it was indicated that the high yields of GM crops were achieved at lower land areas (Burney et al., 2010). The improved productivity also reduces the pressure of converting additional land for agriculture (Bouët and Gruère, 2011). Genetic modification reduces habitat destruction, which is a common practice of intensive farming that poses a large threat to biodiversity. For instances, without the use of GM crops, an additional 22.4 Mha would have been needed for maintaining the global production at 2016 levels (Brookes and Barfoot, 2018).

GM crops are considered as unique species that pose a threat through movement of their genes (Raman, 2017). At present there are no scientific manifestation of hazards associated with the transfer of genes between unrelated organisms developed through genetic alterations. Different scientific corporations such as the U.S. National Academy of Sciences, World Health Organization (WHO), and the British Royal Society have stated that consumption of GM foods is not as harmful as consuming the same foods that were modified using conventional crop improvement techniques. Therefore, the GM crops cannot be prevented for use in sustainable food production systems.

### 6.2 GM crops for sustainable society

The adoption of GM crops has significant health benefits. It reduces the exposure to harmful chemical pesticides that are used with non-GM crops (Smyth, 2020b). Two decades analysis of GM corn consumption by Pellegrino et al. (2018) indicated that it posed no threat to the health of human or livestock. It showed a substantive positive impact on health due to the presence of lower mycotoxins in crops (Pellegrino et al., 2018). The emergence of new genetic modification technologies enabled the production of crop varieties with enhanced flavors and reduced allergens (Mathur et al., 2015). Moreover, the prospective production of edible vaccine in GM crops can result in low-cost vaccine production and allow for their accessibility to a larger section of the society. The pre-testing for the safety of GM crops in several areas has indicated no evidence of any adverse reactions (Kamle et al., 2017). Although the negative health consequences of GM crops consumption are reported on rats, analyses of most of the studies about the safety of GM crops, indicated no human health consequences (Szymczyk et al., 2018; Giraldo et al., 2019).

The sustainable food production systems need to ensure food security for the growing population. Since most of the countries depend on the food imports for their supply’s due to the climatic constraints and the insect pests, food security appears difficult (Xiao et al., 2020). However, GM crops climatic stress tolerance and higher yields will ease the process of achieving food security (Evanega et al., 2022; Keiper and Atanassova, 2022). Therefore, including the GM crops into the sustainable food production systems will enable different communities to produce their own food. Moreover, the GM crops are developed with improved shelf-life that can be stored for longer periods without wastage. Such practices appeal to the ethical principles of beneficence and justice, which means to have fair and equitable food supply that will benefit the larger society (Smyth, 2020a; MatouskovaVanderberg, 2022; Vega Rodríguez et al., 2022).

The genetic modification of crops further provides the different nutrients required for healthy human living. Kettenburg et al. (2018) depicted the evidence of health gains from the Bt maize crops and Golden Rice that produces Vitamin A for human beings. It has been reported that around 1 million children die annually due to the Vitamin A deficiency (Swamy et al., 2019). Therefore, the production of Golden Rice plays an important role in preventing these deaths of children. Hence, the introduction of GM crops can save human lives. The potential risks of GM crops that are not proven remain insignificant for people who are starving or having severe nutrient deficiencies (Vega Rodríguez et al., 2022). People with life threatening disease deploy themselves to experimental drugs, which is considered ethical after a consent, the same could be applied to the GM crops.

The experts from governmental and non-governmental agencies in some of the developing countries have increasingly included the GM crops into the wider approaches of sustainability (Hartline-Grafton and Hassink, 2021). However, there are certain people within different communities who still resist the GM crops because of the personal and religious beliefs (Bawa and Anilakumar, 2013). It includes the concern over the right to “play God”, as well as the introduction of any foreign gene into crops that are abstained for religious reasons (Omobowale et al., 2009). It is believed that it is intrinsically wrong to tamper with nature, and others consider inserting new genes into plant genome as unethical (Daunert et al., 2008). However, such an issue can be addressed through genome editing techniques and with the contrasting view that the genetic modification is simply one more step in the processes of modification of the physical world. It is similar to the manufacturing of novel chemicals in industries and to natural breeding of plants and animals (Yang et al., 2022). As people are having a choice to use different novel chemicals, similarly a right to choose can be developed for the GM crops consumption. Moreover, the science and technology have advanced humans in putting adequate measures to evaluate and monitor scientific innovations to prevent potential risks to the society (John and Babu, 2021). Therefore, it is of support to use GM crops in sustainable food production systems, as the development of GM crops is identical to any another scientific invention.

### 6.3 GM crops for sustainable economy

The economical aspect of GM crops faces the issue of intellectual property rights (Delgado-Zegarra et al., 2022). The producers of GM crops have used terminator technology to protect their seeds and reduce the gene flow. The seeds and pollens of these crops are made sterile (Turnbull et al., 2021). After the completion of harvest, farmers have to re-purchase the seeds from the seed producers. It has been argued that such a technique provides seed companies more control over what the farmers should grow, and it is considered to be unethical by the society (Delgado-Zegarra et al., 2022). But, from an innovation perspective, it is ethical to protect intellectual properties, because these seeds are the obtention of biotechnological companies, they need to have the same intellectual property protection rights as any other potential product, such as the protection of a new software developed by an IT company (Muehlfeld and Wang, 2022). However, it is due to the negative publicity of GM crops that they are held back by the public. There are also very few farmers that depend on second-generation seeds. Hence, the introduction of sterile seeds does not affect the famer’s seed choices (Addae-Frimpomaah et al., 2022). Many of the GM seed manufacturers developed a solution towards sterile seeds through the creation of seed contract with the farmers. The seed contract is an agreement that states that the GM seeds are sterile and are used by farmers on their own choice and that the seeds shouldn’t be distributed for any other purposes. This has resulted in economic benefits to the seed producers in an ethical way through the farmer contract agreement.

The farmers have also benefited economically with the adoption of GM crops (Raman, 2017). With the introduction of GM crops, there would be a major increase in the farmer’s production efficiency which in turn results in higher revenue (Oliver, 2014). Since the GM crops are made resistant to pests, the cost spent on chemical pesticides will decline, as less chemicals will be required for the GM crops (Buiatti et al., 2013). Furthermore, the use of farm machineries declines as well due to no-tillage practices with GM crops that reduces the cost spent on the fuel of machineries. In addition to this, the land cost for the growers can decline using GM crops as these crops produce high yield in small spaces. Moreover, the poor farmers are mostly engaged in subsistence farming, but the adoption of GM crops would enable such farmers to market their products due to surplus yields from GM crops (Azadi et al., 2016), which would improve their quality of life within the society (Lucht, 2015). The farmers have well-adopted GM crops into the food production systems. Since mid-1990s, GM crops were planted by 18 million farmers (ISAAA, 2017). The track records indicated logistical and economic advantages to the farmers. A net economic benefit of USD 186.1 billion within the twenty-one years of GM crop use was recorded in various farms. It was found by Brookes and Barfoot (2018) that 52% of these benefits were reaped by farmers from developing country. The majority of these gains (65%) were mainly due to yield and productivity increases, while the remaining (35%) resulted from the cost savings.

## 7 Conclusion

The practice of sustainable agriculture has become challenging due to the changes in climate, the rising population, and shrinkage of arable lands. There is a need to develop modified crops having higher productivity, quality, and tolerance to various biotic and abiotic stresses. The genetic modification of crops has enabled the development of efficient production systems that provided substantial benefits to the producers and the community based on the three principles of sustainable agriculture such as protecting environment, enhancing human health, and improving the economy.

Even when there are strict assessments of environmental and health safety, and these crops are granted regulatory approval, concerns are still raised over the involvement of the genetic modification tools and their long-term unknown disadvantages on environment and health. The potential negative consequences of GM crops have caused to their lesser implementation in various countries. To overcome and address some of these concerns, new advanced alternative molecular techniques are developed, such as genome editing, particularly CRISPR/Cas9 system that improves crop traits without introducing foreign genes. The expansion of plant breeding to genetic modification through genome editing would further produce more per unit of land that makes them essential in achieving the SDGs, especially for eradicating hunger, improving food security and human health.

The present review indicates that it would be imprudent to dismiss GM crops as a tool for meeting the goals of sustainable development. With the increasing global challenges, GM crops can help humanity. However, it is imperative that the scientific community and agricultural industries invest in better communications and regulations to counter the misinformation and unethical research associated with GM crops. Moreover, this review suggests that GM crops can be broadly adopted by improving the already present regulations, efficient monitoring, and practice implementation through government agriculture bodies. In addition, developing a global risk alleviation strategy and communication with growers, will ensure a substantial acceptance and adoption of GM crops in several countries to bring global profitability and productivity.

Finally, the sustainability of GM crops should be determined based on their role in sustainable agriculture and human development within the next 30 years. It is not only GM crops that pose certain risks and concerns, but all the methods of food production are associated with some drawbacks. However, the use of genome editing tools and regulation of GM crops ensure that these crops are as safe as conventionally bred crops and can act as the drivers of sustainable food security.

## Author contributions

KM and MA conceptualized and wrote the original manuscript. KM, MA, FB, and HR. reviewed and edited the draft paper. All the authors have read and approved to the submitted version of the manuscript.

## Funding

This research work was supported by funding from the United Arab Emirates University, the Research Office to KM under grant number 31R203.

## Conflict of interest

The authors declare that the research was conducted in the absence of any commercial or financial relationships that could be construed as a potential conflict of interest.

## Publisher’s note

All claims expressed in this article are solely those of the authors and do not necessarily represent those of their affiliated organizations, or those of the publisher, the editors and the reviewers. Any product that may be evaluated in this article, or claim that may be made by its manufacturer, is not guaranteed or endorsed by the publisher.
